# Oleate induces K_ATP_ channel-dependent hyperpolarization in mouse hypothalamic glucose-excited neurons without altering cellular energy charge

**DOI:** 10.1016/j.neuroscience.2016.12.053

**Published:** 2017-03-27

**Authors:** Selma Dadak, Craig Beall, Julia M. Vlachaki Walker, Marc P.M. Soutar, Rory J. McCrimmon, Michael L.J. Ashford

**Affiliations:** a Division of Molecular and Clinical Medicine, School of Medicine, Ninewells Hospital and Medical School, University of Dundee, Dundee DD1 9SY, UK; b Institute of Biomedical and Clinical Sciences, University of Exeter Medical School, RILD Building, Barrack Road, Exeter EX2 5DW, UK

**Keywords:** ACC, acetyl-CoA carboxylase, AMPK, adenosine 5′-monophophate-activated protein kinase, AMP-PNP, 5′-adenylylimidodiphosphate, ARC, arcuate nucleus, CPT1, carnitine palmitolytransferase-1, CSF, cerebrospinal fluid, GE, glucose-excited, GI, glucose-inhibited, K_ATP_, ATP-sensitive potassium channel, oleate, POMC, proopiomelanocortin, UCP, uncoupling protein, VMN, ventromedial nucleus, glucose sensing, K_ATP_, oleate, hypothalamus, mitochondria, fatty acid oxidation

## Abstract

The unsaturated fatty acid, oleate exhibits anorexigenic properties reducing food intake and hepatic glucose output. However, its mechanism of action in the hypothalamus has not been fully determined. This study investigated the effects of oleate and glucose on GT1-7 mouse hypothalamic cells (a model of glucose-excited (GE) neurons) and mouse arcuate nucleus (ARC) neurons. Whole-cell and perforated patch-clamp recordings, immunoblotting and cell energy status measures were used to investigate oleate- and glucose-sensing properties of mouse hypothalamic neurons. Oleate or lowered glucose concentration caused hyperpolarization and inhibition of firing of GT1-7 cells by the activation of ATP-sensitive K^+^ channels (K_ATP_). This effect of oleate was not dependent on fatty acid oxidation or raised AMP-activated protein kinase activity or prevented by the presence of the UCP2 inhibitor genipin. Oleate did not alter intracellular calcium, indicating that CD36/fatty acid translocase may not play a role. However, oleate activation of K_ATP_ may require ATP metabolism. The short-chain fatty acid octanoate was unable to replicate the actions of oleate on GT1-7 cells. Although oleate decreased GT1-7 cell mitochondrial membrane potential there was no change in total cellular ATP or ATP/ADP ratios. Perforated patch and whole-cell recordings from mouse hypothalamic slices demonstrated that oleate hyperpolarized a subpopulation of ARC GE neurons by K_ATP_ activation. Additionally, in a separate small population of ARC neurons, oleate application or lowered glucose concentration caused membrane depolarization. In conclusion, oleate induces K_ATP-_dependent hyperpolarization and inhibition of firing of a subgroup of GE hypothalamic neurons without altering cellular energy charge.

## Highlights

• Oleate and low glucose hyperpolarize and inhibit GT1-7 and mouse GE neurons by activation of K_ATP_.
• Oleate inhibition of GT1-7 neuron activity is not mediated by AMPK or fatty acid oxidation.
• Activation of K_ATP_ by oleate requires ATP hydrolysis but does not reduce the levels ATP or the ATP:ADP ratio.
• GT1-7 hyperpolarization by oleate is not dependent on UCP2.
• Oleate and low glucose depolarize a subpopulation of hypothalamic GI neurons.

## Introduction

The hypothalamus is critical for the continuous regulation of whole-body glucose, lipid and energy homeostasis. To perform this function, various hypothalamic nuclei (e.g. arcuate (ARC), ventromedial (VMN), lateral hypothalamic area and paraventricular) contain neuropeptide-expressing neurons that monitor circulating nutrients and hormone levels (Levin et al., 2004). Scattered throughout these nuclei are subpopulations of neurons that sense changes in glucose levels, resulting in altered neuronal firing and modified energy homeostasis (Levin et al., 1999). There are two main subtypes of glucose-sensing hypothalamic neurons that contribute to these homeostatic mechanisms: neurons excited (glucose-excited (GE)) and inhibited (glucose-inhibited (GI)) by increased levels of glucose. GE neurons utilize ATP-sensitive potassium (K_ATP_) channels (in a manner similar to pancreatic beta cells (MacDonald et al., 2005) to modulate their electrical activity in response to changes in extracellular glucose concentration (Ashford et al., 1990, Wang et al., 2004, Kang et al., 2006). It is presently unclear which ion transport mechanism is responsible for transducing changes in glucose concentration to modify electrical activity in GI neurons (Gonzalez et al., 2009). These glucose-sensing neurons, in particular GE neurons, play important roles in the feeding response to glucoprivation (as suppression of glucokinase (GK) diminishes the glucoprivic stimulation of feeding (Dunn-Meynell et al., 2009), and liver glucose production (Parton et al., 2007) and have been strongly implicated in the detection of hypoglycemia and subsequent generation of counterregulatory responses (Beall et al., 2012). For example, loss of the K_ATP_ channel subunit, K_IR_6.2, causes near complete suppression of glucagon responses to hypoglycemia, which is driven by the loss of K_ATP_ on neural cells (Miki et al., 2001).

Similarly, energy status (i.e. lipid level) is also communicated continuously to the hypothalamus. This is performed, at least in part, by hypothalamic neurons responding to changes in the concentrations of circulating hormones (e.g. leptin and insulin), the levels of which correlate with adipose tissue depot size. It is this latter communication system that is considered to become faulty in obesity (Frederich et al., 1995). However, circulating lipids, such as long-chain fatty acids, have also been demonstrated to directly act on hypothalamic centers to modulate feeding and hepatic glucose output (Obici et al., 2002, Lam et al., 2005). Circulating levels can be acutely elevated, such as during fasting or hypoglycemia, where lipolysis is elevated (De Feo et al., 1989), therefore it is plausible that some neurons have a capacity to detect both reduced glucose and elevated lipid levels. Indeed the long-chain fatty acid, oleate alters ARC neuron neuropeptide expression, electrical activity and within glucosensing neurons, can alter intracellular calcium signaling via CD36/fatty acid translocase (Obici et al., 2002, Morgan et al., 2004, Wang et al., 2006, Le Foll et al., 2009). Furthermore, long-chain fatty acids are activators of K_ATP_ channels in pancreatic beta cells (Larsson et al., 1996) and hypothalamic-delivered oleate suppresses hepatic glucose production in a K_ATP_-dependent manner (Obici et al., 2002). A previous study suggested that oleate alters the excitability of ARC neurons in a glucose-concentration-dependent manner suggesting interaction at the level of cellular nutrient metabolism (Wang et al., 2006). In addition, the central effects of fatty acids on glucose homeostasis have been ascribed to neuronal fatty acid metabolism (Obici et al., 2003, Cruciani-Guglielmacci et al., 2004).

Alternatively, fatty acids can regulate the activity of mitochondrial uncoupling proteins (UCP) (Echtay et al., 2001), including the neuronal enriched UCP isoforms, UCP4 and UCP5 (Hoang et al., 2012). In the pancreatic beta cell, UCP2 has been implicated in regulating glucose-sensing behavior (Lameloise, 2001, Zhang et al., 2001, Parton et al., 2007). Indeed, oleate modulates the expression of UCP2 and alters glucose-dependent insulin secretion in pancreatic beta cells and beta cell lines (Medvedev et al., 2002, Oprescu et al., 2007). Importantly, UCP2 has been shown to regulate the glucose-sensing behavior of GE neurons and alter whole-body glucose homeostasis (Parton et al., 2007). Consequently, in order to explore the mechanisms by which oleate modifies neuronal excitability we have examined the interplay between nutrient-dependent pathways and oleate responses in mouse GE-type neurons.

## Experimental procedures

### Cell culture

Immortalized mouse hypothalamic GnRH secreting GT1-7 cells (Pamela Mellon, San Diego, California, USA (Lam et al., 2005) were maintained in Dulbecco’s modified Eagle’s medium (DMEM; Sigma–Aldrich, Gillingham, UK)) with 10% fetal bovine serum (PAA Laboratories, Yeovil, UK & Hyclone, Pasching, Austria) as described (Mirshamsi et al., 2004).

### Immunoblotting

GT1-7 cells, seeded in 6-well dishes, were serum-starved for three hours with DMEM replaced by saline and then treated with nutrients (0–100 μM oleate or 2.5/0.1 mM glucose) for various times as described in the results section. We used oleate concentrations in the low micromolar (10–100 μM) as plasma concentrations of total free fatty acids have been estimated within the range of 350–500 μM under normal circumstances (Richieri and Kleinfeld, 1995, Mai et al., 2006, Abdul-Ghani et al., 2008) and, in type 1 diabetes, can reach levels of more than 1200 μM (Boden, 1998). In human CSF levels of oleate have been reported to be >10 μM in non-diabetic populations (Levi et al., 2013), and may be expected to reach much higher levels in type 1 diabetic patients. In rats, brain glucose levels have been estimated at ∼2.5 and ∼0.1 mM during euglycemia and hypoglycemia, respectively (Silver and Erecinska, 1994).

Protein isolation and immunoblotting procedures were as described previously (Mirshamsi et al., 2004). Briefly, protein lysates were subjected to SDS–PAGE, electrotransferred to nitrocellulose membranes, and probed with primary antibodies against p-AMPKα (Thr172; 1:1000), p-ACC (Ser79; 1:1000), actin (1:5000), UCP4 (1:1000) and UCP5 (1:1000). All antibodies were obtained from Cell Signalling Technology Inc. (New England Biolabs, Hitchin, UK) except UCP4 and UCP5, which were obtained from Acris Antibodies (Herford, Germany). Proteins were detected with horseradish peroxidase-conjugated Goat anti-Rabbit IgG and immunoreactive proteins identified by chemiluminescence. Gel protein bands were quantified by densitometry, where total density was determined with respect to constant area, background subtracted and average relative band density calculated.

### Hypothalamic slice preparation

All animal procedures conformed to the UK Animals Scientific Procedures Act (1986) and were approved by the University of Dundee institutional ethics review committee. Wild-type male C57Bl/6 mice (6–20 weeks old) were killed by cervical dislocation and the brains rapidly removed and submerged in an ice cold slicing solution as described previously (Hisadome et al., 2009). Briefly, hypothalamic coronal slices containing the ARC (350 μm) were prepared using a Vibratome (St Louis, MO, USA) and stored at room temperature (22–25 °C) in an external solution containing (in mM): NaCl 125, KCl 2.5, NaH_2_PO_4_ 1.25, NaHCO_3_ 25, CaCl_2_ 2, MgCl_2_ 1, D-Glucose 10, D-Mannitol 15, ascorbate 1 and pyruvate 3, equilibrated with 95% O_2_, 5% CO_2_, pH 7.4. Immediately before use, brain slices were transferred to the recording chamber of an upright Zeiss Axioskop-2 FS plus microscope and continuously perfused with a modified external solution (containing 0.5 mM CaCl_2_ and 2.5 mM MgCl_2_, no ascorbate and pyruvate) at a constant flow rate of 5–10 ml min^−1^ and bath temperature of 33 °C.

### Electrophysiology

GT1-7 cells were visualized by phase contrast and individual neurons of mouse hypothalamic slices by differential interference contrast optics. Whole-cell patch-clamp recordings were performed using borosilicate pipettes (4–8 MΩ) filled with an internal solution containing (mM): K-gluconate 130, KCl 10, EGTA 0.5, HEPES 10, NaCl 1, MgCl_2_ 3, CaCl_2_ 0.28, Na_2_-ATP 3, Tris-GTP 0.3, and phosphocreatine 14; pH 7.2 for ARC neurons and with KCl 140, EGTA 10, HEPES 10, MgCl_2_ 5, CaCl_2_ 3.8, and Na_2_-ATP 3; pH 7.2 for GT1-7 cells. Hypothalamic neuron recordings were made in external solution at 33 °C and GT1-7 cell recordings at room temperature (22–25 °C) in saline containing (in mM): NaCl 135, KCl 5, MgCl_2_ 1, CaCl_2_ 1, HEPES 10, glucose 2.5 (pH 7.4). For perforated patch-clamp recordings 30–35 μg/ml amphotericin B and/or 2.5 mg/ml gramicidin was included in the internal solution in the absence of Na_2_-ATP. As previously described, voltage and current commands were manually or externally driven using pCLAMP 9.2 software and injected into neurons via the patch-clamp amplifier (Axopatch 200B; Molecular Devices, Sunnyvale CA, USA). In the whole-cell current-clamp configuration, hyperpolarising current pulses (5–20 pA amplitude at a frequency of 0.05 Hz) were used to monitor changes in input resistance. For voltage-clamp recordings the membrane potential was held at −70 mV and voltage steps (20 mV increments, 200 ms duration) applied over the voltage range of −160 to −20 or +80 mV. Voltage-clamp protocols were carried out immediately after whole-cell formation, prior to and during exposure to drugs and/or oleate or altered glucose concentration (following achievement of maximal responses). At least 10 min of stable control data were recorded before the application of drugs or oleate, which were added to the external/saline solution and applied to slices or GT1-7 cells via a superfusion system. In some slice experiments oleate was applied locally via pressure ejection using a broken-tipped pipette positioned above the recording neuron.

### Nucleotide measurements

GT1-7 cells were seeded onto black-walled 96-well plates at 1 × 10^4^ cells per well. Total ATP measurements were made using ATPLite assay kit (Perkin-Elmer, Seer Green, UK), as per the manufacturer’s protocol, with minor modifications. Briefly, cells were lyzed with shaking, using 40 μl of mammalian cell lysis buffer for 10 min at 700 rpm, followed by the addition of 40 μl of ATP substrate solution. For ATP/ADP ratios, cells were plated as for total ATP measurements. Ratios were determined using an ATP/ADP ratio kit (Sigma–Aldrich, Gillingham, UK) as per the manufacturer’s instructions.

### Mitochondrial membrane potential

GT1-7 cells were seeded on to glass-bottom dishes (In Vitro Scientific, CA, USA) 24 h prior to study. JC-1 was used as per the manufacturer’s protocol with minor modifications. Briefly, cells were pre-incubated with 2 μg/ml JC-1 for 1 h at 37 °C in a 95% O_2_/5% CO_2_ humidified incubator before washing twice with saline containing 10 mM glucose and studied immediately. Images were captured on a Leica SP5 laser scanning confocal microscope and fluorescence intensity analyzed using the standard Leica LAS AF software and GraphPad Prism 5 for statistical analyses.

### Non-ratiometric calcium imaging

GT1-7 cells were seeding into 96-well plates at 2 × 10^5^ cells per well. Cells were loaded with Fluo4 Direct (ThermoFisher, UK) for 1 h at 37 degrees. Fluorescence was monitored using a Pheratar FS multifunction plate reader (BMG Labtech, Aylesbury, Buckinghamshire, UK), with oleate added by injection and cells maintained at 37 degrees during the imaging protocol. Fluorescence is presented as relative fluorescent units (RFU), with fluorescence at the zero point being normalized to 1.

### Chemicals

Amphotericin B, AMP-PNP, FA-free bovine serum albumin, diazoxide, etomoxir, gramicidin, malonyl-CoA, octanoic acid, oleic acid, and tolbutamide were purchased from Sigma–Aldrich. Compound C was obtained from Merck Chemicals Ltd (Nottingham, UK). Genipin was purchased from Wako Chemicals (Eastleigh, UK) and JC-1 was obtained from Thermo Fisher Scientific (Renfrew, UK). Oleate was prepared as a 10 mM stock solution containing 5% NEFA-free BSA.

### Statistics

In slice recordings, hypothalamic neurons responding to drugs, oleate or altered glucose concentration were distinguished from non-responding neurons based on the criterion that the change in membrane potential (ΔVm) induced by the challenge was ± three times the standard deviation of the mean membrane potential prior to the challenge. Results are expressed as the mean ± S.E.M. of the defined responses, with the number of cells studied. Statistical significance was determined by Student’s *t*-test or ANOVA followed by Bonferroni’s post hoc test where appropriate. Changes in protein phosphorylation in Western blot experiments were analyzed using a one-sample *t*-test for multiple comparisons with respect to control. A *P* value of less than 0.05 was considered statistically significant.

## Results

### Mouse hypothalamic GT1-7 cells exhibit GE behavior and are oleate-sensitive

Attempting to study the mechanism(s) by which oleate alters ARC neuron excitability is difficult when recording from unidentified neurons in a hypothalamic slice. Therefore, we utilized the mouse hypothalamic cell line, GT1-7, which exhibits GE-type properties. We have previously demonstrated that this neuronal cell line shows graded electrical responses over a physiological range of glucose concentrations and that these responses can be modulated by pharmacological manipulation of the classical components of glucose-sensing, namely glucokinase, adenosine 5′-monophosphate-activated protein kinase (AMPK) and K_ATP_ (Beall et al., 2012). They therefore represent an excellent model for studying the mechanisms through which oleate may alter neuron excitability.

**Fig. 1:**
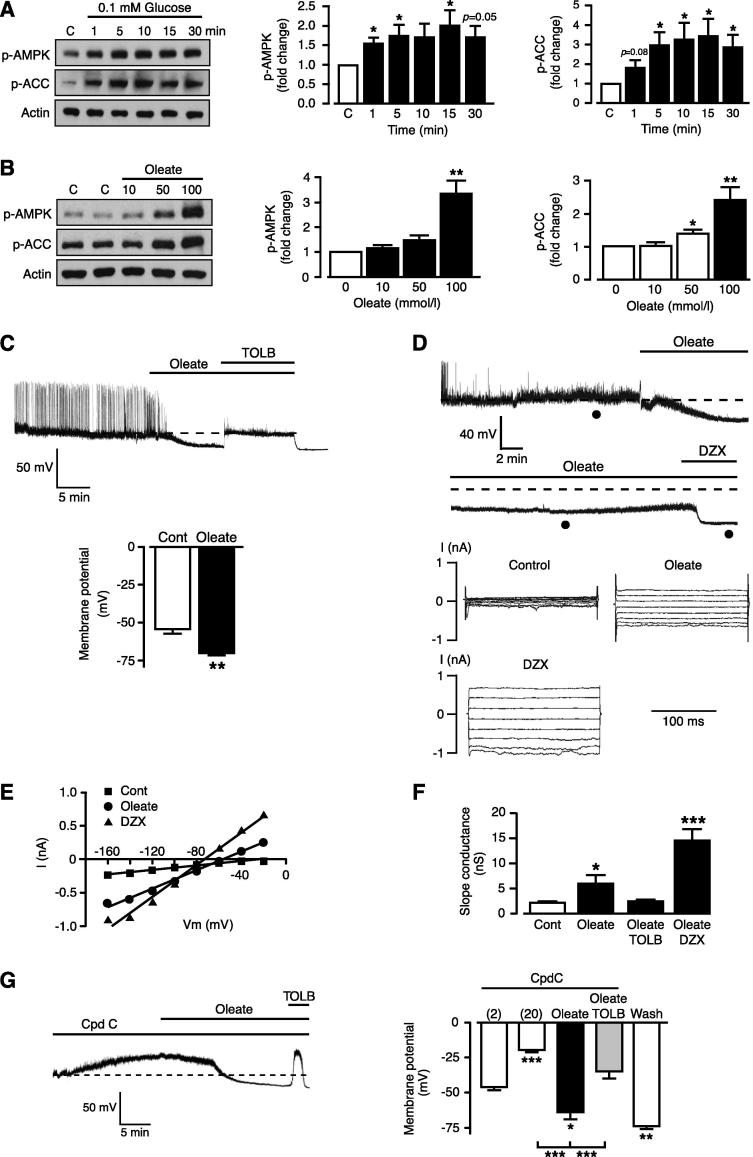
Oleate activates AMPK and K_ATP_ in glucose-sensing GT1-7 neurons. (A) Representative immunoblots showing the effect of lowering glucose concentration from 2.0 to 0.1 mM on p-AMPK and p-ACC levels with time. Bar graphs show relative mean levels of p-AMPK and p-ACC as a function of time after challenge with 0.1 mM glucose (*n* = 6). (B) Representative immunoblots showing the effect of increasing concentration of oleate (10, 50 and 100 μM) on the levels of p-AMPK and p-ACC. Bar graphs show relative mean levels of p-AMPK and p-ACC as a function of oleate concentration (*n* = 5–8). (C) Oleate hyperpolarizes GT1-7 cells within 5–15 min of application, an action reversed on application of tolbutamide. Bar graph shows mean values for membrane potential of cells exposed to oleate (*n* = 3). (D) Whole-cell recording under current-clamp and switched to voltage-clamp for short periods as denoted by (●) showing that the oleate (100 μM) hyperpolarization is sustained but submaximal with addition of diazoxide (250 μM) causing further hyperpolarization (upper traces). The families of currents shown are from a holding potential of −70 mV with tests pulses between −160 and −20 mV and obtained at times marked (●) during the experiment shown in upper traces. (E) Current–voltage relationships formed with current amplitude values from the cell shown in (D). (F) Bar graph denotes mean slope conductance values (derived from best fit to current–voltage curves over −160 to −20 mV range) in control and after addition of 100 μM oleate, oleate + tolbutamide and oleate + Dzx (*n* = 6). (G) Effect of oleate (100 μM) on the membrane potential of a GT1-7 cell in which compound C (20 μM) was present in the electrode solution and allowed to dialyze into the cell for 20 min prior to oleate application. Note that oleate hyperpolarized the cell, which was reversed by the addition of tolbutamide (200 μM). Bar graph shows mean values for membrane potential of cells dialyzed with compound C ∼2 min after formation of whole-cell configuration and after 20-min dialysis, followed by the addition of oleate, tolbutamide and extensive wash (*n* = 4–7). Values are mean ± SEM. ^*^p < 0.05, ^**^p < 0.01, ^***^p < 0.001

In GT1-7 cells, a hypoglycemic challenge (0.1 mM glucose) of 30 min resulted in increased AMPK phosphorylation (p-AMPK) (Fig.1A). This was accompanied by increased acetyl-CoA carboxylase (ACC) phosphorylation (p-ACC), a key substrate of AMPK, indicating increased AMPK activity. As AMPK has been implicated as a key component of cellular glucose sensing in hypothalamic neurons (Claret et al., 2007), GT1-7 neurons (Beall et al., 2012) and pancreatic beta cells (Beall et al., 2010), we next examined whether oleate also alters AMPK activity. Oleate (10–100 μM; Fig.1B) increased levels of AMPK and ACC phosphorylation with a minimum oleate concentration to elicit this response of >50 μM (Fig.1B). Thus, oleate mimics the effects of lowered glucose levels on p-AMPK/p-ACC levels in GT1-7 cells over a similar time course. Consequently, we next determined whether oleate also induced cell hyperpolarisation and K_ATP_ activation in GT1-7 cells as demonstrated previously for lowered glucose concentrations (Beall et al., 2012).

Hence we performed perforated patch recordings (which minimizes disruption of normal cell glucose metabolism) from GT1-7 cells to monitor changes in electrical activity and firing frequency. Application of oleate (100 μM) to GT1-7 cells induced hyperpolarization and inhibition of firing (Fig.1C). Baseline membrane potential was −54.3 ± 3.1 mV, which hyperpolarized to −70.0 ± 1.5 mV following approximately 15 min of oleate treatment (*n* = 3, *P* < 0.05), and reversed by application of tolbutamide (200 μM) suggesting activation of K_ATP_ channels. The lowest concentration at which oleate induced a detectable hyperpolarization of GT1-7 cells was ∼50 μM (data not shown). To determine the contribution of K_ATP_, we utilized whole-cell voltage-clamp, intermittent with current-clamp recordings to examine the effect of oleate on K_ATP_ currents following attainment of the maximal hyperpolarizing response. Firstly, oleate hyperpolarized GT1-7 neurons (Fig.1D upper traces) with a similar time course to that observed in perforated patch recordings. Secondly, this hyperpolarization was accompanied by an increased total current (Fig.1D lower traces), a shift in the reversal potential from −29.2 ± 6.5 mV to −58.9 ± 4.5 mV (*n* = 6; *P* < 0.01; Fig.1E) and an increased slope conductance (Fig.1F). The increase in conductance elicited by oleate was returned to control levels by concomitant application of 200 μM tolbutamide (*n* = 3; Fig.1F). Additionally, GT1-7 cells under voltage-clamp exposed to 100 μM oleate and subsequently challenged by addition of the direct K_ATP_ channel opener diazoxide (250 μM) in the continued presence of oleate, demonstrated further cell hyperpolarization (Fig.1D), an additional negative shift of the reversal potential (to −76.0 ± 2.2 mV; *n* = 6; *P* < 0.01; Fig.1E) with an enlarged current associated with a greater slope conductance (Fig.1D, F). These actions of oleate are consistent with submaximal activation of K_ATP_. In order to assess whether the increase in AMPK activity drives the oleate-mediated hyperpolarization and K_ATP_ activation, we determined the effect of oleate on whole-cell recordings with the AMPK inhibitor (Zhou et al., 2001), compound C (20 μM) in the pipette solution. The presence of compound C resulted in a significantly more depolarized membrane potential for GT1-7 cells, in comparison to untreated control cells, but did not prevent oleate from hyperpolarizing the cells in a tolbutamide-dependent manner (Fig.1G). Consequently, these data suggest that, in contrast to lowered glucose level (Hisadome et al., 2009), elevated AMPK activity is not required for oleate-mediated activation of K_ATP_ channels and cell hyperpolarization.

### GT1-7 cell response to OA is independent of fatty acid oxidation

**Fig. 2:**
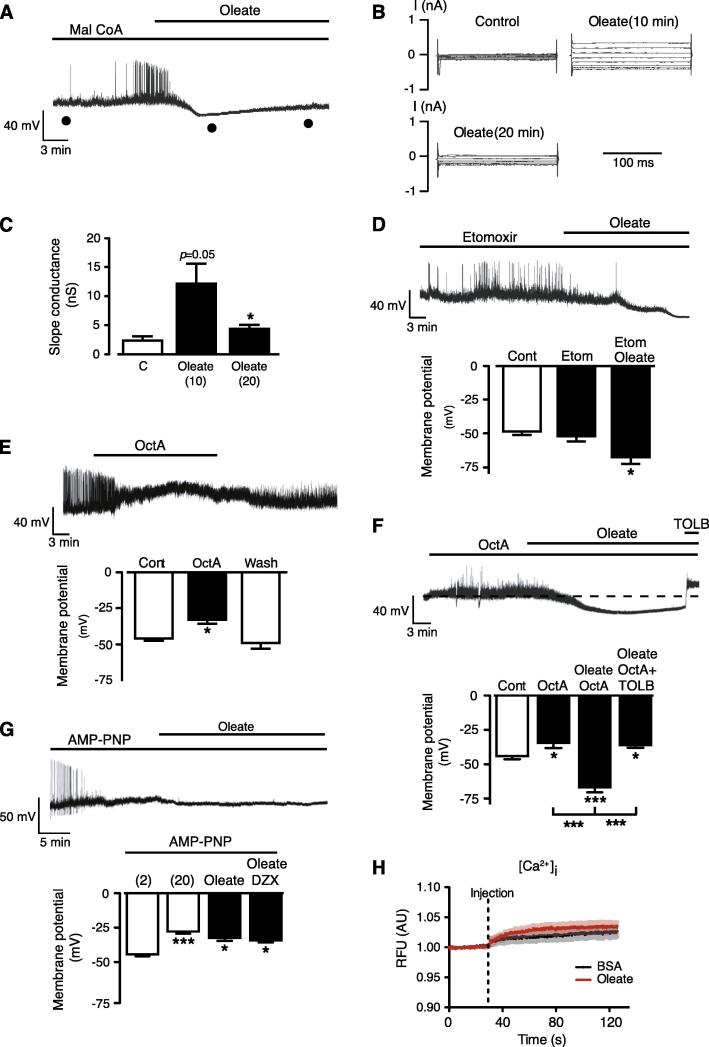
K_ATP_ activation by oleate is independent of AMPK, fatty acid oxidation and not mimicked by octanoic acid. (A) Effect of oleate (100 μM) on membrane potential of a GT1-7 cell in which malonyl-CoA (50 μM) was present in the electrode solution and allowed to dialyze into the cell for 20 min prior to oleate application. (B) Families of currents shown are from a holding potential of −70 mV with tests pulses between −160 and −20 mV and obtained at the marked times (●) during the experiment shown in (A). (C) Bar graph denotes mean slope conductance values (derived from best fit to current–voltage curves over −160 to −20 mV range) in control and in the presence of 100 μM oleate (*n* = 4), 10 and 20 min after oleate addition. (D) Effect of oleate (100 μM) on membrane potential of a GT1-7 cell in which etomoxir (100 μM) was present in the electrode solution and allowed to dialyze into the cell for 20 min prior to oleate application. Bar graph shows mean values for membrane potential of untreated (concurrently performed) cells (Cont), etomoxir (Etom) and Etom + oleate (*n* = 4). (E) Octanoic acid (50 μM) reversibly depolarizes GT1-7 cell membrane potential. Bar graph shows mean values for membrane potential of cells exposed to control (Cont), octanoic acid (OctA) and following extensive washout of OctA (*n* = 8). (F) Oleate (100 μM) hyperpolarizes GT1-7 cells in the presence of OctA (50 μM), an action reversed by tolbutamide (200 μM). Bar graph shows mean values for membrane potential of cells exposed to control (Cont), octanoic acid (OctA), OctA + oleate and OctA + oleate + Tolb (*n* = 3–4). (G) The presence of AMP-PNP (3 mM) in the electrode solution (and thus dialyzed into cell) resulted in cell depolarization and prevented hyperpolarization by oleate (100 μM) and DZX (250 μM). Bar graph shows mean values for membrane potential of cells exposed to control (Cont: ∼2 min after formation of whole-cell configuration and after 20-min dialysis, followed by the addition of oleate and oleate + DZX (*n* = 4). (H). Calcium imaging traces following addition of BSA (control) of oleate (100 μM; *n* = 5). Values are means ± SEM. ^*^p < 0.05, ^***^p < 0.001

It has previously been reported that oleate induces depolarization and excitation of mouse ARC proopiomelanocortin (POMC) neurons via mitochondrial β-oxidation, raised ATP concentration and subsequent inactivation of K_ATP_ channel activity (Jo et al., 2009). As GT1-7 cells respond to oleate by activation of K_ATP_ and cell hyperpolarization such a mechanism was unlikely to also explain this outcome. Alternatively, increased fatty acid oxidation could raise reactive oxygen species levels (Ruiz-Ramírez et al., 2011, Arany et al., 2013, Gremmels et al., 2015) previously implicated in pathways leading to neuronal K_ATP_ activation (Chai and Lin, 2010). Thus in order to examine the role of fatty acid metabolism via β-oxidation we prevented the delivery of oleate to mitochondria by inhibition of carnitine palmitoyltransferase-1 (CPT1). Whole-cell recordings were used to deliver directly to the interior of the cell (drug present in patch pipette solution), either malonyl-CoA (50 μM) an endogenous CPT1 inhibitor (McGarry et al., 1977) or etoxomir (100 μM) an irreversible CPT1 inhibitor (Declercq et al., 1987). The presence of malonyl-CoA did not significantly alter the membrane potential of GT1-7 cells (−43.9 ± 2.3 mV; *n* = 10; *P* > 0.05 vs untreated controls), and subsequent challenge with oleate resulted in membrane potential hyperpolarization and increased K_ATP_ conductance (Fig.2A–C). The actions of oleate in the presence of malonyl-CoA were similar to that of control experiments (above), however, although the initial (5–10 min) response to oleate in the presence of malonyl-CoA was indistinguishable from control, we did note that the oleate response waned with time and by 20 min was reduced by >50% (Fig.2A–C). This outcome could indicate a delayed reduction in fatty acid oxidation by malonyl-CoA, mediated perhaps by diffusion limitations. Thus, in separate experiments, we examined the effect of etomoxir (applied via pipette solution with a dialysis time of at least 20 min prior to oleate challenge) and showed that this drug had no effect *per se* on the resting membrane potential of GT1-7 cells and did not prevent oleate from causing cell hyperpolarization (Fig.2D). Consequently, it appears unlikely that β-oxidation of oleate is required to elicit the inhibition of GT1-7 cell electrical activity. We next examined whether the short-chain fatty acid octanoate (C8), which does not require CPT1-dependent transport into the mitochondria for oxidation, could mimic the hyperpolarizing effect of oleate on GT1-7 cells. Surprisingly, addition of octanoate (50 μM) to GT1-7 cells caused a significant depolarization, which was reversible on washout of the fatty acid (Fig.2E). However, the presence of this short-chain fatty acid did not prevent oleate from hyperpolarizing GT1-7 cells in a tolbutamide-dependent manner (Fig.2F). These results indicate that GT1-7 cells respond by different effector mechanisms to short- and long-chain fatty acids. In calcium imaging studies, we found that oleate treatment did not alter intracellular calcium (Fig.2H) suggesting that CD36 is not involved in this response.

### Oleate hyperpolarization of GT1-7 cells may be dependent on ATP metabolism

Previously it has been suggested that oleate may inhibit ATP-dependent gating of an acetylcholine-modulated potassium channel (Kim and Pleumsamran, 2000). As K_ATP_ channel activity is controlled to a large extent by the ATP/ADP ratio in cells, with ATP-inhibiting and ADP-activating K_ATP_ activity, respectively (Tucker and Ashcroft, 1998), a simple explanation for the oleate-mediated hyperpolarization could be reduced ATP-dependent inhibition of channel activity. To examine this possibility, we first decided to replace ATP with the non-hydrolyzable ATP analog, 5′-adenylylimidodiphosphate (AMP-PNP; 3 mM) in the pipette solution. The mean GT1-7 cell resting membrane potential was significantly depolarized by the presence of AMP-PNP (Fig.2G), compared to control experiments in which ATP was allowed to wash into the cell interior (compare with Fig.2A). Furthermore, neither oleate (up to 200 μM) nor diazoxide (250 μM) application to cells dialyzed with AMP-PNP resulted in hyperpolarization of the membrane potential (Fig.2G), conflicting with the idea that oleate may act to displace ATP from its inhibitory site on K_ATP_. However, these data suggest the requirement for ATP hydrolysis for K_ATP_ activation, as previously demonstrated for diazoxide in beta cells (Larsson et al., 1993).

### Oleate reduced JC-1 mitochondrial membrane potential fluorescence without altering cellular energy charge

**Fig. 3:**
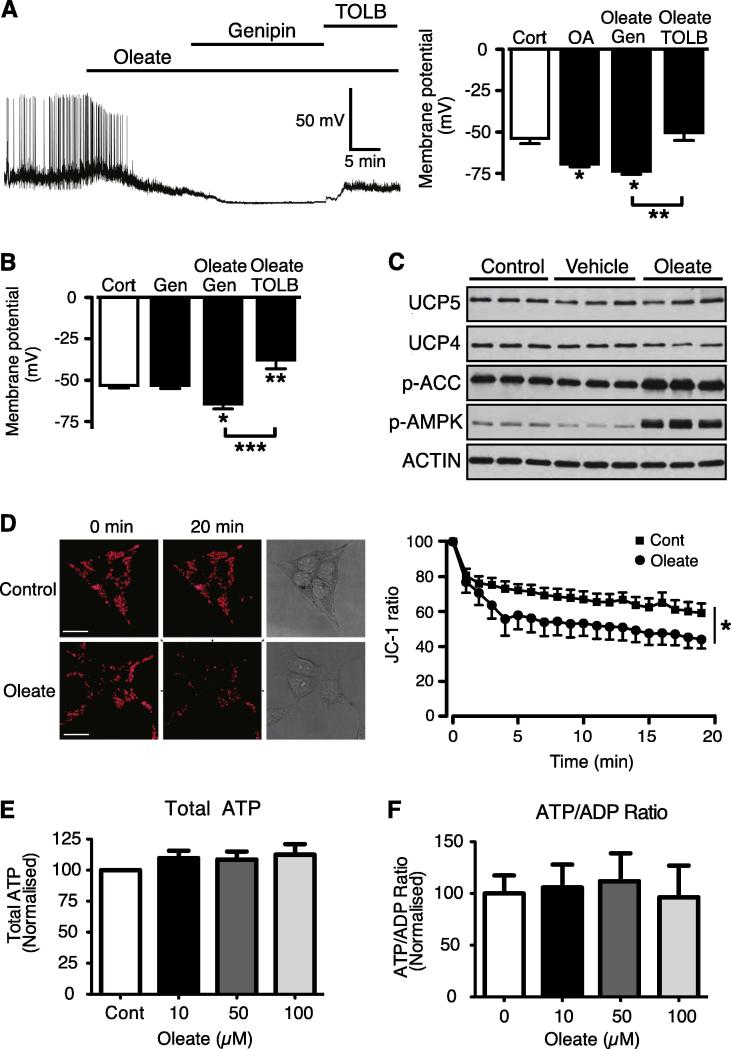
Oleate hyperpolarizes GT1-7 neurons independent of UCP2 activity but depolarizes the mitochondrial membrane potential. (A) Representative perforated patch recording from a GT1-7 neuron showing that 100 μM oleate-induced hyperpolarization is unaffected by the presence of 100 μM genipin, and depolarized by subsequent application of 200 μM tolbutamide. Bar graph shows mean values for membrane potential of cells before and after application of oleate, followed by oleate + genipin and oleate + tolbutamide (*n* = 4). (B) Bar graph showing data from similar experiment to (A), but with 100 μM genipin added prior to challenge with oleate + genipin, followed by oleate + tolbutamide (*n* = 3). (C) Representative immunoblots showing the levels of UCP5, UCP4, p-ACC, p-AMPK and actin for control, vehicle (BSA) and oleate-treated cells. (D) Confocal images of GT1-7 cells pre-incubated with JC-1 under control conditions and treated with oleate (100 μM) for 20 min. Conventional light images of cells are shown on the right panels. Graph showing the change in JC-1 fluorescence intensity with time (relative to starting point of recording) for control (filled squares) and oleate-treated cells (*n* = 8). (E) GT1-7 total cellular ATP levels and (F) ATP:ADP ratios are unchanged by the addition of oleate (10, 50 and 100 μM). Values are mean ± SEM. ^*^p < 0.05, ^***^p < 0.001

Previous studies have indicated an important role for uncoupling protein-2 (UCP2) as a regulator of K_ATP_ channels in glucose-sensing neurons (Parton et al., 2007), and oleate has been demonstrated to increase levels of UCP2 in beta cells (Medvedev et al., 2002). Increasing cellular UCP2 levels and activity would be expected to diminish mitochondrial membrane potential and respiration, via uncoupling, and so reduce ATP levels, which could provide an explanation for oleate-mediated activation of K_ATP_ in these neurons. We examined this possibility by inhibiting UCP2 activity pharmacologically with genipin (Zhang et al., 2006), which has previously been shown to reverse low glucose-mediated hyperpolarization of pancreatic beta cells, GE-type hypothalamic neurons and GT1-7 cells (Parton et al., 2007, Beall et al., 2010, Beall et al., 2012). In contrast we found that genipin was unable to reverse, or prevent, oleate from hyperpolarizing GT1-7 neurons (Fig.3A, B) suggesting that UCP2 activity is not mediating oleate action on these cells. However, on screening GT1-7 cells for additional UCP isoforms we found that they also express UCP4 and UCP5, although their protein levels were not altered by oleate treatment (Fig.3C). Consequently, to explore further whether uncoupling by oleate was a possible mechanism we assessed GT1-7 mitochondrial membrane potential using the membrane permeant mitochondrial dye, JC-1. Oleate significantly decreased JC-1 fluorescence indicating depolarization of the mitochondrial membrane potential (Fig.3D) in a time course that mimics oleate-mediated changes in membrane potential and K_ATP_ current in these cells. To determine whether the oleate-induced mitochondrial depolarization reduced cellular energy charge, we measured both total cellular ATP and the ATP/ADP ratio. Neither measure was significantly altered by exposure to increasing concentrations of oleate (Fig.3E, F), further indicating that the activation of K_ATP_ channels by oleate is unlikely to be caused by reduced ATP-mediated inhibition.

### ARC neurons exhibit oleate and glucose sensitivity

**Fig. 4:**
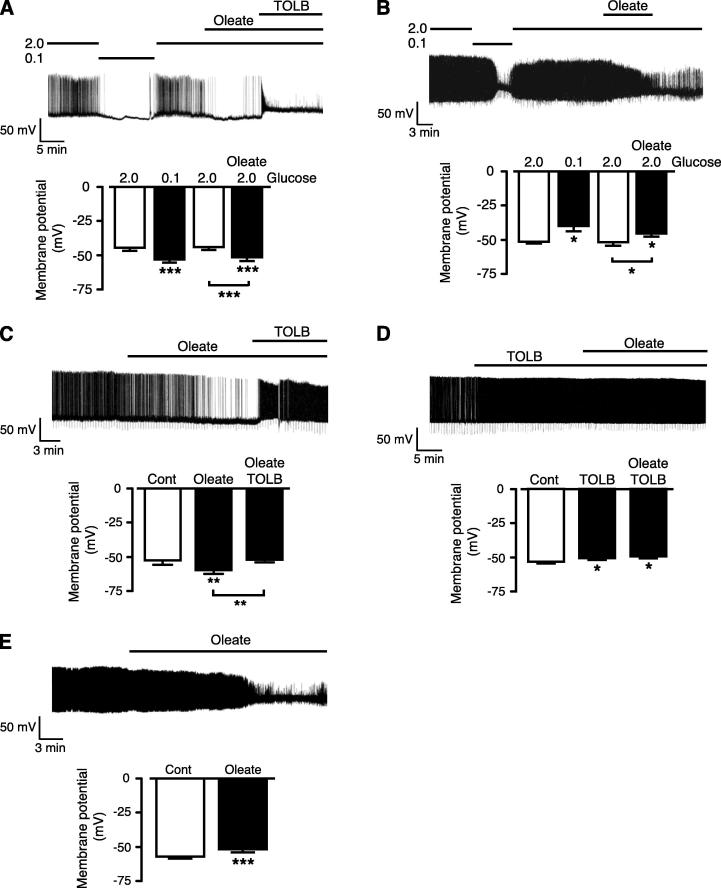
Subpopulations of mouse ARC neurons respond electrically to oleate. (A) Representative perforated patch recording from a GE neuron, as demonstrated by the reversible hyperpolarization elicited on switch from 2 mM to 0.1 mM glucose. Subsequent application of oleate (100 μM) in the presence of 2 mM glucose hyperpolarizes this GE neuron. Addition of tolbutamide (200 μM) in the presence of oleate depolarizes the neuron. Note the loss of action potentials in the presence of oleate + TOLB. Bar graph shows mean values (*n* = 6) for membrane potential of neurons exposed to 2.0 mM and 0.1 mM glucose and 2 mM glucose + oleate. (B) Representative perforated patch recording from a GI neuron, as demonstrated by the reversible depolarization elicited on switch from 2 mM to 0.1 mM glucose. Subsequent application of oleate (100 μM) in the presence of 2 mM glucose depolarizes this GI neuron. Bar graph shows mean values (*n* = 4) for membrane potential of neurons exposed to 2.0 mM and 0.1 mM glucose and 2.0 mM glucose + oleate. (C) Representative whole-cell current-clamp recording from an unidentified ARC neuron, with application of 100 μM oleate producing hyperpolarization of the membrane potential and inhibition of firing, actions reversed by concomitant addition of tolbutamide (200 μM). Bar graph shows mean values (*n* = 4) for membrane potential of neurons exposed to: Control (Cont), 100 μM oleate and tolbutamide (TOLB). (D) Tolbutamide (200 μM) induced depolarization and increased firing in an unidentified ARC neuron (indicating the presence of active K_ATP_ channels) under whole-cell current-clamp. Subsequent addition of oleate (100 μM) had no effect on membrane potential or firing rate. Bar graph shows mean values (*n* = 15) for membrane potential of neurons exposed to: Cont, TOLB and oleate + TOLB. (E) Representative whole-cell current-clamp recording from an unidentified ARC neuron demonstrating the addition of oleate (100 μM) depolarized some neurons with an overall decrease in firing and truncation of action potential amplitude. Bar graph shows mean values (*n* = 13) for membrane potential of neurons in Cont and exposed to oleate. Values are mean ± SEM. ^*^p < 0.05, ^**^p < 0.01, ^***^p < 0.001

We then sought to translate our findings in GT1-7 cells to acute brain slice preparations from mice. First we performed perforated patch recordings on unidentified ARC neurons using a physiological extracellular glucose concentration (2 mM) (Silver and Erecinska, 1994, de Vries et al., 2003), similar to the recording conditions for the GT1-7 cells. We used hypoglycemic challenge (0.1 mM glucose) and subsequent changes in membrane potential and firing rates to identify GE- and GI-type neurons, prior to challenge with oleate. Thus, when GE ARC neurons were challenged with 0.1 mM glucose, they responded, by hyperpolarization (from a Vm of −46.6 ± 1.2 mV to −53.8 ± 1.5 mV (*n* = 20); *P* < 0.01), which was reversible on return to 2 mM glucose solution (Fig.4A). Subsequent addition of 100 μM oleate resulted in hyperpolarization (ΔVm = −7.0 ± 1.1 mV; *P* < 0.01) and cessation of firing in six out of 13 GE neurons tested, with concomitant application of tolbutamide (200 μM) reversing the hyperpolarization caused by the presence of oleate. The remaining GE neurons were unaffected by oleate (data not shown). Interestingly, in ARC neurons that responded, reversibly, to 0.1 mM glucose by depolarization (from a Vm of −49.3 ± 0.8 mV to −38.9 ± 1.7 mV (*n* = 13; *P* < 0.01)) and were thus deemed GI-type, subsequent application of 100 μM oleate exhibited depolarization (ΔVm = +6.4 ± 1.2 mV) in four out of seven GI neurons tested (Fig.4B), with the remaining GI neurons unaffected (data not shown). Neurons that displayed no change in membrane potential or firing rate to 0.1 mM glucose challenge were similarly electrically unresponsive to oleate (*n* = 5; data not shown). Consequently, subpopulations of glucose-sensing GE and GI ARC neurons respond to oleate, with the change in electrical excitability mirroring the effect of hypoglycemic levels of glucose.

As performed for GT1-7 cells, we also tested the effect of oleate on the electrical activity of mouse unidentified ARC neurons using the whole-cell current-clamp recording configuration. Under these conditions the mean resting membrane potential for ARC neurons was −52.9 ± 0.5 mV (*n* = 51), similar to that reported previously for this recording mode in hypothalamic neurons (Song et al., 2001, Ibrahim et al., 2003, Claret et al., 2007). Bath application or pressure ejection of oleate (100 μM) resulted in hyperpolarization (Fig.4C), with a mean ΔVm of −6.9 ± 0.6 mV (*n* = 32; *P* < 0.001) and inhibition of firing from 4.1 ± 0.4 Hz to 1.6 ± 0.4 Hz (*P* < 0.001). This inhibitory response was accompanied by a decrease in the mean input resistance from 2.13 ± 0.15 GΩ to 1.87 ± 0.16 GΩ (*P* < 0.05), indicative of an increased cell conductance. Application of tolbutamide (200 μM) in the continued presence of oleate, reversed the hyperpolarization and inhibition of firing (Fig.4C; *n* = 4; *P* < 0.01). In addition, naïve ARC neurons that responded with a small depolarization (ΔVm = +2.8 ± 1.1 mV; *n* = 15; *P* < 0.05) and increased firing to the application of 200 μM tolbutamide (indicating the presence of active K_ATP_ channels and so likely to be GE-type) were shown to be unaffected by addition of 100 μM oleate in the continued presence of tolbutamide (Fig.4D). These data are in general agreement with the GT1-7 findings and indicate that oleate can inhibit neuronal firing by activation of K_ATP_ channels in at least a subpopulation of hypothalamic GE neurons. In a small number of ARC neurons under these whole-cell recording conditions we found that oleate caused depolarization (ΔVm = +5.1 ± 0.7 mV; *n* = 6; *P* < 0.01), which resulted in no significant change in firing frequency (4.4 ± 1.4 Hz to 6.4 ± 0.8 Hz; *n* = 6; *P* = 0.09), possibly because the depolarization was sufficient in some cases to result in inactivation of voltage-gated sodium/calcium channels and inhibit action potential generation (Fig.4E).

## Discussion

Previous studies have produced disparate outcomes on whether lipid-sensing hypothalamic neurons are also glucose sensing. Wang and colleagues (Wang et al., 2006) showed that there is minimal overlap between oleate-sensing and glucose-sensing neurons in rat ARC whereas Le Foll et al. (2009) demonstrated, using rat VMN neurons, that many GE- and GI-type neurons were excited and inhibited, respectively, by oleate (Wang et al., 2006, Le Foll et al., 2009). We find that glucose-sensing ARC neurons are also oleate sensing in the same direction with respect to change in excitability. Thus GE neurons are inhibited by exposure to oleate and by lowered extracellular glucose concentration, via a K_ATP_-dependent mechanism. Similarly, ARC neurons depolarized by oleate are also depolarized by lowered extracellular glucose concentration. These data indicate that for these mouse ARC neurons at least, there is a shared cellular response to increased lipid availability and glucose deprivation. Thus it is likely that such neurons play important roles in the modulation of physiological responses to the fed-fasted transition and/or in the pathophysiological outcomes associated with hypoglycemia and/or starvation. Both insulin-induced hypoglycemia and starvation are associated with increased lipolysis (De Feo et al., 1989). However, lipid levels are also elevated in the post-prandial period therefore perhaps the glucose level stratifies the physiological response to increased lipid availability. Therefore, it is possible that moderate changes in lipid availability converge on these neuron populations to amplify an energy deficit signal or to enhance hepatic glucose production, depending on glucose availability.

To investigate the intracellular mechanism(s) responsible for oleate sensing in hypothalamic GE neurons we utilized the mouse hypothalamic GnRH-releasing GT1-7 neuronal cell line, which we have recently characterized as a GE-type glucose-sensing neuron (Beall et al., 2012), with glucose-sensing properties similar to that described for ARC neurons, including POMC and AgRP neurons (Claret et al., 2007) and GnRH neurons (Zhang et al., 2007). Challenging GT1-7s with oleate elicited hyperpolarization and reduction or cessation of firing, which was reversed or prevented by tolbutamide, indicating a K_ATP_-dependent mechanism, similar to that observed in ARC GE neurons under the same perforated patch-recording conditions. As this outcome was observed on isolated GT1-7 cells, this suggests that the oleate response is intrinsic to neurons and cell autonomous. We have previously shown that low glucose-induced opening of K_ATP_ in mouse pancreatic beta cells, POMC neurons and GT1-7 neurons is dependent on AMPK activity (Claret et al., 2007, Beall et al., 2010, Beall et al., 2012). Although oleate increases AMPK activity in GT1-7 cells (similar to that observed for low-glucose stimulation), the oleate-induced hyperpolarization of GT1-7 cells was not prevented by the presence of the AMPK inhibitor, compound C. Thus oleate-driven modulation of K_ATP_ channel activity in GT1-7 neurons is likely mediated by a mechanism distinct from that of hypoglycemia.

Interestingly, the oleate-induced neuronal hyperpolarization reported here is in contrast to a previous study, which demonstrated that oleate depolarized mouse ARC POMC neurons, which are GE-type neurons (Ibrahim et al., 2003), by inhibition of K_ATP_ channels, an outcome ascribed to increased mitochondrial beta-oxidation of oleate and raised cellular ATP levels (Jo et al., 2009). An alternative mechanism by which mitochondrial oxidation of oleate could increase K_ATP_ activity and cause cell hyperpolarization is via the enhanced production of reactive oxygen species (Larsson et al., 1996, Arany et al., 2013, Gremmels et al., 2015). Importantly, the effects of oleate on energy and glucose homeostasis have been shown to be dependent on hypothalamic CPT1 activity (Obici et al., 2003, Pocai et al., 2006), indicative of fatty acid oxidation regulating hypothalamic neurons. CPT1 is endogenously inhibited by malonyl-CoA, the product of the reaction carried out by acetyl CoA carboxylase. However, we found that oleate-induced GT1-7 neuron hyperpolarization was not altered by direct intracellular application of malonyl-CoA or by etomoxir, a pharmacological inhibitor of CPT1, suggesting that oleate-induced K_ATP_ channel activation and subsequent hyperpolarization does not require mitochondrial fatty acid metabolism. Furthermore, the short-chain fatty acid, octanoic acid induced a significant depolarization of GT1-7 cells, but did not block the hyperpolarizing action of oleate. Moreover, oleate did not alter intracellular calcium in the GT1-7 cells, suggesting that the oleate-induced changes are unlikely to be mediated by CD36-driven store calcium release (Le Foll et al., 2009) or conventional PKCs. Consequently, there is no evidence to indicate that an alteration in fatty acid beta-oxidation or fatty acid transport via CD36 is involved in the oleate-mediated hyperpolarization of hypothalamic neurons. Perhaps this is unsurprising as mitochondrial FA metabolism predominantly generates ATP, which would be expected to maintain K_ATP_ channel closure, as described previously (Jo et al., 2009).

It has recently been suggested that coupling of the extracellular glucose concentration to K_ATP_ in pancreatic β-cells and GT1-7 neurons may also be dependent on the expression and activity of UCP2 (Beall et al., 2010, Beall et al., 2012). UCP2 acts to dissipate the proton gradient across the mitochondrial membrane and is expressed throughout the VMH (Parton et al., 2007) and in GT1-7 cells (Beall et al., 2012). Furthermore, UCP2 is activated by long-chain fatty acids (Jabůrek, 1999), including oleate (Lameloise, 2001, Zackova et al., 2003) or indirectly via fatty acid-induced production of reactive oxygen species such as superoxide (Krauss et al., 2003) or lipid peroxidation intermediates (Murphy et al., 2003, Malingriaux et al., 2013). Thus oleate activation of UCP2 is expected to decrease the mitochondrial membrane potential and mitochondrial respiration, resulting in reduction of cellular ATP levels. Acute pharmacological inhibition of UCP2 with genipin did not prevent nor reverse the oleate-induced hyperpolarization of GT1-7 neurons, indicating that UCP2 does not play a role in oleate-induced changes in membrane potential. Nonetheless, indirect measurement of the mitochondrial membrane potential indicated that oleate induced a mild uncoupling of the mitochondrial membrane potential in a time course that correlated with the activation of K_ATP_. This led us to screen GT1-7 neurons for other UCP isoforms, with UCP4 and UCP5, the brain-enriched isoforms (Sanchis et al., 1998, Mao et al., 1999), which display proton transport capacity within lipid membranes (Hoang et al., 2012). Both UCP4 and UCP5 were detected by immunoblotting, although their expression was not significantly modified over the time course of the oleate treatment. Importantly however, the reduced JC-1 fluorescence caused by oleate did not translate to a reduction in either total ATP levels or a change in the ATP/ADP ratio. Therefore it is possible that oleate quenched the JC-1 fluorescence in a non-specific manner (Mathur et al., 2000, Perry et al., 2011) and that none of the UCP isoforms are involved in oleate-induced K_ATP_ channel opening.

This leaves open the question of what intracellular mechanism(s) mediates oleate-induced K_ATP_ channel activation and hyperpolarization of GT1-7 and ARC GE neurons. Since ATP levels are not altered and oleate hyperpolarizes GE neurons and GT1-7 cells (where ATP levels are clamped to maintain K_ATP_ channels closed), it is unlikely that activation is mediated by a deficit in ATP availability. It is possible that oleate may reduce ATP availability in the microdomain around the K_ATP_ channel that is not detectable with whole-cell ATP measurements, or that oleate (or a metabolite) alters K_ATP_ ATP sensitivity or displaces ATP from the nucleotide binding domain of the channel. An alternative possibility is that oleate is converted to a long-chain fatty acyl CoA (LC–CoA) in neurons. This process requires ATP hydrolysis and LC–CoA molecules have been demonstrated to activate K_ATP_ by binding to a specific site on the channel (Larsson et al., 1996, Bränström et al., 1997).

There are a number of limitations to our studies. Firstly, we have not been able to address the mechanism of oleate-induced depolarization in GI ARC neurons, in large part due to the lack of a suitable cell culture model. Therefore, the current that underlies hypoglycemic and oleate-induced neuronal depolarization remains elusive. A recent mouse model developed by Stanley and colleagues (Stanley et al., 2013) allowing identification of GI neurons for slice electrophysiology may be useful for identifying this current.

The concentration of oleate used here is larger than that previously used in some studies of hypothalamic glucose-sensing neurons (Wang et al., 2006, Le Foll et al., 2009), although a recent study in humans measured an oleate level of >10 μM in the CSF of non-obese humans (Levi et al., 2013). Furthermore, our data demonstrating phosphorylation (inhibition) of ACC in response to 100 μM oleate corresponds with that previously shown to inhibit ACC and fatty acid synthesis in astrocytes (Natali et al., 2007). Therefore the concentration used here probably lies in the upper end of the physiological level or in the pathophysiological range seen in type 1 diabetes (Boden and G., 1998) and/or obesity. However, there may be a number of reasons for the discrepancies in oleate sensitivity. Previous studies have utilized acutely dissociated VMH neurons, the preparation of which may induce a stress response sufficient to alter the sensitivity of these neurons to alternate fuel sources. Secondly, we have used whole-cell and perforated patch-clamp studies to measure whole-cell macroscopic currents, membrane potential and firing frequency whereas other studies have utilized Ca^2+^ imaging. Indeed, oleate can induce changes in intracellular calcium by mobilizing [Ca^2+^]_i_ stores (Carrillo et al., 2011), which may change independently of cellular membrane potential, although we did not observed in the current study.

The ability of oleate to reproduce the effects of low glucose on glucose-sensing neurons in the hypothalamus might be expected to drive food intake and increase hepatic glucose production since direct pharmacological opening of hypothalamic K_ATP_ channels drives glucagon and adrenaline release in the periphery during hypoglycemia (Evans et al., 2004). However, central oleate (under normoglycemic conditions) has been shown to inhibit feeding and suppress glucose production, (Obici et al., 2002, Cintra et al., 2012). This is further complicated by the observation that the effect of oleate on feeding and glucose production requires the activity of K_ATP_ (Obici et al., 2002), indicating that the action of oleate on feeding and glucose production may utilize a similar intracellular mechanism described here. To examine this relation in more detail, it would be interesting in future experiments to examine changes in ARC neuropeptide ratios (NPY/POMC and AgRP/POMC) and counterregulatory hormone responses to insulin-induced hypoglycemia under conditions of high (oleate infusion) or low (acipimox-treated) plasma fatty acid levels. It is plausible that separate subpopulations of ARC neurons, which are involved in discrete physiological functions (Lam et al., 2015, Burke et al., 2016) respond differentially to oleate (by depolarization, hyperpolarization or no response). Further analysis of electrophysiological responses of identified ARC neurons to oleate and other common dietary fatty acids, in conjunction with mouse metabolic phenotypic analysis is required to resolve this issue. Moreover, our studies did not examine the influence of hormones on low glucose and oleate responses. It is plausible that leptin, insulin and ghrelin, for example, modulate oleate and glucose responses, depending on the physiological context of energy surfeit or deficit.

In summary, we have demonstrated that the long-chain unsaturated fatty acid, oleate alters the activity of glucose-sensing hypothalamic neurons in a manner similar to that of a hypoglycemic stimulus. Furthermore, by utilizing a hypothalamic glucose-sensing cell line, we show that oleate-induced K_ATP_ channel activation occurs independently of changes in energy charge and may involve an oleate metabolite that directly alters K_ATP_ channel activity. The assimilation of fatty acid- and glucose-sensing neuronal populations, along with the influence of hormones such as insulin and leptin are likely to be important drivers of an integrated physiological response to changing nutrient availability. Clearly, additional research is required to delineate the network integration of glucose- and fatty acid-sensing neurons within the hypothalamus and their relation to energy homeostasis.

## Author contributions

M.L.J.A. conceived the study. S.D., C.B., J.V.W., M.P.M.S, R.J.M. and M.L.J.A. contributed to experimental design, analysis of data and writing of the manuscript. S.D. and C.B. performed the patch-clamp studies and S.D. and M.P.M.S. performed the Western blotting studies and C.B. performed the imaging studies. J.V.W. performed the nucleotide measurements and calcium imaging studies. All authors read and approved the final manuscript.
